# Nonlinear resonance-assisted tunneling induced by microcavity deformation

**DOI:** 10.1038/srep09010

**Published:** 2015-03-11

**Authors:** Hojeong Kwak, Younghoon Shin, Songky Moon, Sang-Bum Lee, Juhee Yang, Kyungwon An

**Affiliations:** 1 School of Physics and Astronomy, Seoul National University, Seoul 151-742, Korea; 2 Korea Research Institute of Standards and Science, Daejeon 305-340, Korea; 3 Russia Science Seoul, Korea Electrotechnology Research Institute, Seoul 121-912, Korea

## Abstract

Noncircular two-dimensional microcavities support directional output and strong confinement of light, making them suitable for various photonics applications. It is now of primary interest to control the interactions among the cavity modes since novel functionality and enhanced light-matter coupling can be realized through intermode interactions. However, the interaction Hamiltonian induced by cavity deformation is basically unknown, limiting practical utilization of intermode interactions. Here we present the first experimental observation of resonance-assisted tunneling in a deformed two-dimensional microcavity. It is this tunneling mechanism that induces strong inter-mode interactions in mixed phase space as their strength can be directly obtained from a separatrix area in the phase space of intracavity ray dynamics. A selection rule for strong interactions is also found in terms of angular quantum numbers. Our findings, applicable to other physical systems in mixed phase space, make the interaction control more accessible.

Asymmetrically deformed microcavities (ADM's) made of dielectric material can serve as versatile platform in photonics applications. Coming in various shapes such as ellipse, quadrupole, stadium, Limaçon and rounded triangle, they can provide directional output^1^,^2^,^3^,^4^,^5^,^6^,^7^,^8^,^9^,^10^, increased energy storage^11^ and enhanced output coupling efficiencies^3^, which are preferred features for micro- and nano-photonics devices. Recently, deformation-induced interactions among cavity modes have also drawn much interest in search of new functionality and enhanced light-matter coupling. From the interaction between an isotropic high-*Q* mode and a directional low-*Q* mode, a new mode can be engineered with the desired assets, high-*Q* and good directionality^7^,^12^. Intermode interactions can also lead to topological singular points called the exceptional points^13^, the unusual properties of which have recently been much investigated as in divergent Petermann factor^14^,^15^,^16^ for enhance photoemission, single particle sensors^17^ and nontrivial lasing threshold and reversed pump dependence^18^,^19^.

The analysis of intermode interactions in ADM's have been mostly performed through numerically solving the wave equations in case-by-case basis. It is because the Hamiltonian responsible for the interactions is completely unknown in those deformed microcavities. There has been no practical method to predict the strength of intermode interaction beforehand. Meanwhile, theoretical advances on interstate interactions have been made for abstract objects such as quantum maps^20^,^21^,^22^,^23^,^24^, where a fictitious Hamiltonian can be constructed to calculate the interaction strength by using the theory of the resonance assisted tunneling (RAT)^23^.

RAT is one type of the dynamical tunneling, a quantum-mechanical tunneling phenomenon to occur between dynamically separated classical trajectories^25^. RAT is a universal phenomenon expected to occur in any weak-perturbed systems of near-integrable or mixed phase space since the theory of RAT does not depend on the details of the Hamiltonian. In contrast to the chaos-assisted tunneling^26^,^27^,^28^,^29^,^30^,^31^,^32^,^33^ mediated by chaotic sea, RAT is enhanced by the presence of nonlinear resonances between regular trajectories. The concept of RAT was initially employed in physical chemistry^34^ for explaining vibrational level splittings. It has also been studied in one-dimensional time periodic quantum maps such as the kicked Harper model and the kicked rotor^20^,^21^,^22^,^23^,^24^. RAT theory has then been employed in analyzing a wide range of physical systems such as periodic-driven pendula^35^, Rydberg atoms under periodic perturbation^36^, quantum accelerator modes^37^ and multi-dimensional molecules^38^,^39^. Despite a large number of theoretical studies on RAT, however, there have been no experiments yet directly verifying the RAT theory.

Here in this paper we report the first experimental observation of RAT in intermode interactions in a weakly deformed two-dimensional microcavity. We examine the strong interactions between two unperturbed-basis modes (UBM's) (Supplementary Note 2). We observe that their interaction strength is proportional to the square of the separatrix area of the phase-space nonlinear resonance chain involved in the interaction. Strong interactions then occur when UBM's satisfy a certain selection rule, namely that their angular mode numbers differ by an integer multiple of the number of islands in the associated nonlinear resonance chain. Furthermore, the proportionality constant or the prefactor is found to depend only on the nonlinear resonance indices. These findings are definitive evidences for RAT. Our results provide a practical way to predict the intermode interactions in a broad range of physical systems in mixed phase space, making the interaction control more accessible.

In order to understand how RAT comes about in ADM's, let us briefly recapitulate the RAT theory. In an integrable multi-dimensional system, classical trajectories appear as invariant tori on the Poincarè surface of section (PSOS), a phase-space representation of classical motion. The Husimi functions, the phase-space projections of quantum eigenstates, are then localized along these tori. In the presence of perturbation, invariant tori are deformed following the Kolmogorov-Arnold-Moser (KAM) scenario and some orbits evolve into a *chain*-like nonlinear resonance structure. According to the RAT theory, a nonlinear resonance structure can then strongly enhance a tunneling process between the UBM's localized along nearby invariant tori when specific conditions are satisfied (see Fig. 1 for illustration). This type of enhanced dynamical tunneling is called RAT^20^,^21^,^40^.

In the RAT theory, an effective Hamiltonian describing the motion near nonlinear resonances can be derived by means of the secular perturbation theory. In a two-dimensional system, the Hamiltonian can be decomposed as
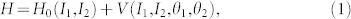
in terms of action-angle variables {*θ_i_*, *I_i_*}, where *H*_0_ is an integrable Hamiltonian and *V* is a perturbation. A resonance arises when 
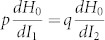
 for co-prime positive integers *p* and *q*, referred to as resonance indices below. Following the standard secular perturbation theory, we can then derive a pendulum-like effective Hamiltonian near the *p*:*q* resonance as
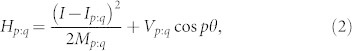
where *I* = *I*_1_, 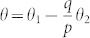, 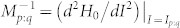 with *I_p_*_:*q*_ the action at the resonance (see Supplementary Note 1 for derivation details). The amplitude *V_p_*_:*q*_ characterizes the coupling strength between eigenstates of the integrable Hamiltonian *H*_0_. The effective Hamiltonian results in a *p*-resonance chain, a chain-like structure of *p* islands in the phase space.

Weakly deformed two-dimensional (2D) microcavities are nothing but weakly perturbed 2D systems and thus the RAT theory can be applied. The Hamiltonian can be expressed in the form of Eq. (1) although we do not know the exact form of *V* (*I*_1_, *I*_2_, *θ*_1_, *θ*_2_). Hence, the effective Hamiltonian is also given by Eq. (2). This identification immediately leads to two important predictions.

First, the interaction term in Eq (2) suggests a *selection rule* that UBM of an angular mode number *m* can be strongly coupled to another UBM of an angular mode number *m* + *i* × *p* (*i* integer)^20^,^21^,^23^ with a strength proportional to 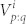 (Supplementary Note 3) in ADM's with small deformation. Second, we can make a connection between the classical phase space and the perturbative amplitude or the coupling strength *V_p_*_:*q*_. Consider a phase space constructed with the action angle variables *I* and *θ*, which are sin *χ* and *s* in Fig. 1, respectively. We can easily calculate the area *S_p_*_:*q*_ enclosed by the separatrix associated with the (*p*:*q*) nonlinear resonance chain, as illustrated in Fig. 1. The result is
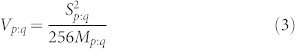
(see Supplementary Note 3 for the derivation). The implication of this result is far reaching. For ADM's, we now have an interaction Hamiltonian given by the second term, *V_p_*_:*q*_ cos *pθ*, in Eq. (2) with its coupling strength *V_p_*_:*q*_ given by the classical phase space or more specifically the PSOS, which is easily obtained by ray tracing. Only quantity we do not know is the prefactor *M_p_*_:*q*_. However, the prefactor can be measured in an experiment as to be discussed below. It can then be used to predict the coupling strength for other intermode interactions as long as they are mediated by the same resonance chain.

## Results

### Design of experiment

The specific physical system we consider is a 2D-ADM made of a liquid jet column of ethanol (refractive index *n* = 1.357) doped with laser dye styryl (LDS) molecules as fluorophore. The details of our liquid jet microcavity are described in Methods (also in Ref. 41). In short, the cavity boundary shape is approximately a quadru-octapole given by 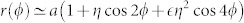, where 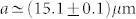 the mean radius and 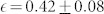. The deformation parameter *η* can be continuously tuned from 0 to 26% by changing the jet ejection pressure.

In order to figure out the spectral regions to investigate in experiments beforehand, it is necessary to survey the interactions among UBM's in our system in numerical simulations. We employed the boundary element method^42^ and calculated the quasi-eigenvalues and associated Husimi functions for the same size and shape as our liquid-jet microcavity. The real part of the quasi-eigenvalues are presented in terms of the size parameter *ka* with *k* = 2*π*/*λ* the wavevector. In our system, the size parameter is inversely proportional to the so-called effective Planck constant 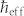 as 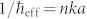.

Figure 2 shows intermode dynamics when *η* = 0.10. For this, we first numerically find high-*Q* mode spectra in the range from 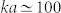 to 180 and identify *uncoupled* mode groups labeled by *radial* mode order *l* (= 1, 2, 3, 4) in the increasing order of their free spectral ranges in an *uncoupled* region (marked by a yellow bar) around *ka* ~ 133. We then define a sequence of reference frequencies of a regular spacing and measure the relative frequencies Δ(*ka*) of each mode group with respect to the reference frequencies. The relative frequencies of all four mode groups are plotted in the mode dynamics diagram in Fig. 2. Detailed information on the uncoupled mode labeling and the relative frequency measurement is described elsewhere^43^.

Each mode group in Fig. 2 more or less follows a *diabatic* line unless it encounters other mode groups. Diabatic lines are shown as dashed lines with associated *l* values denoted in Fig. 2. When mode groups encounter each other, they exhibit avoided crossings (AC's). The AC gap – defined as the smallest energy separation of two interacting levels or mode groups – is approximately twice the coupling strength between them (see below for more explanation). By inspecting the AC gap, we can qualitatively identify two types of interactions, *strong* (circled red) vs. *weak* (not circled) interactions.

### Selection rule

To verify the existence of any selection rule for these interactions, we need to know the angular mode numbers *m*'s of the UBM's associated with the interacting quasi-eigenmodes and compare their difference Δ*m* with the number of islands *p* in the related resonance chain structure. We can infer the angular mode number *m* by inspecting the spatial mode distribution of the quasi-eigenmode in the uncoupled regions (Supplementary Figure 1). In each mode group, *m* increases by 1 when we move up in *ka* by one free spectral range along the diabatic line in Fig. 2. The radial mode number *l*, also called the mode order, of the associated UBM can be identified by counting the number of anti-nodes in the radial direction. We can also identify the resonance chain (thus *p*) involved in the interaction by comparing the PSOS and the Husimi functions of the quasi-eigenmodes in the uncoupled region (Supplementary Figure 2).

The result of our examination on the relation between Δ*m* and *p* in several strong- and weak-interaction cases is summarized in Table I. For all of the strong interaction cases in Fig. 2, the angular mode number difference Δ*m* is equal to the number *p* of the islands in the resonance chain structure as projected by the selection rule in the RAT theory. This is not the case for the weak interaction between *l* = 1 and 3 modes (between *l* = 1 and 4 modes) since the resonance index *p* of 6 (8) is not divisors of the observed Δ*m* of 14 (20). On the other hand, the seemingly weak interaction between *l* = 1 and 2 (*l* = 2 and 4) modes satisfies the selection rule. In fact, the interaction is also induced by RAT, but as shown in Supplementary Note 3, the coupling 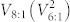 itself is small, resulting in a weak interaction.

### Evaluation of interaction strength

For verification of the relation between the coupling strength and the separatrix area, we measured the AC gap of *l* = 2 and 3 unperturbed modes for various cavity deformation by using the cavity-modified fluorescence spectroscopy^44^. The cavity medium was doped with LDS 821 molecules at a concentration of 0.03 mM/L, covering a spectral range around 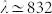 nm (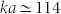). The cavity deformation *η* was varied from 0.065 to 0.12. A representative spectra is shown in Fig. 3a, where among four different mode groups *l* = 2 and 3 modes exhibit an AC with its gap *δV* indicated when *η* = 0.089.

**Figure 1:**
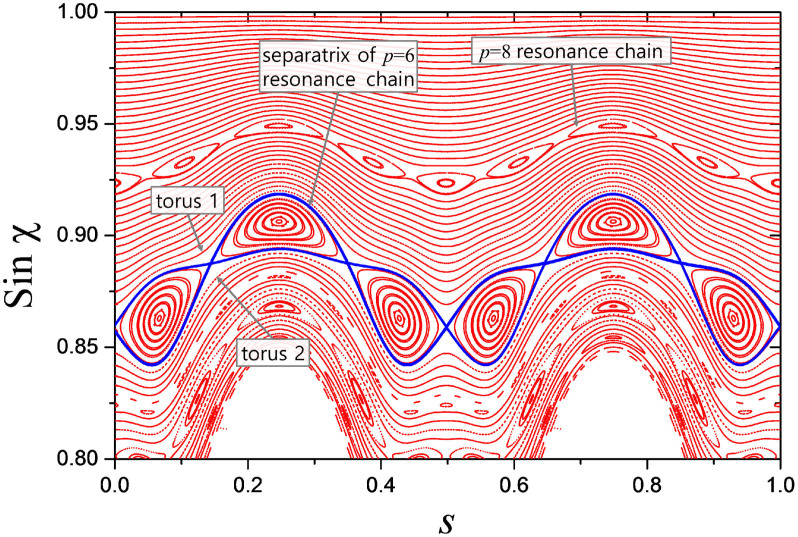
Nonlinear resonance chains in the phase space. For our 2D-ADM to be discussed below, PSOS is constructed in terms of action angle variables *s* and sin *χ*, where a ray is reflected off the cavity boundary with an incidence angle *χ* at the normalized arc-length coordinate *s* (0 ≤ *s* ≤ 1) along the boundary from the major axis. Cavity deformation is given by a parameter *η* = 0.1. Nonlinear resonance chains with *p* = 6 and 8 are easily noticed. The separatrix of the *p* = 6 resonance chain is illustrated. KAM tori 1 and 2 associated with two UBM's are indicated around *p* = 6 resonance chain. RAT can then occur between these UBM's mediated by the *p* = 6 resonance chain.

**Figure 2:**
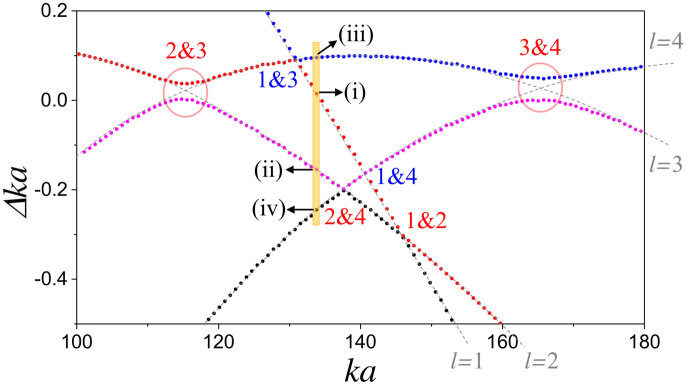
Mode dynamics diagram in our ADM. Relative frequencies Δ(*ka*) of *l* = 1, 2, 3 and 4 modes are plotted with respect to a reference frequency in the range from *ka* ~ 100 to 180 when *η* = 0.10 in our ADM. The AC between *l* = 2 and 3 modes around *ka* ~ 114 is investigated in detail in our experiment. Spatial mode distributions as well as Husimi functions of the modes marked as (i) ~ (iv) are presented in Supplementary Note 4.

**Figure 3:**
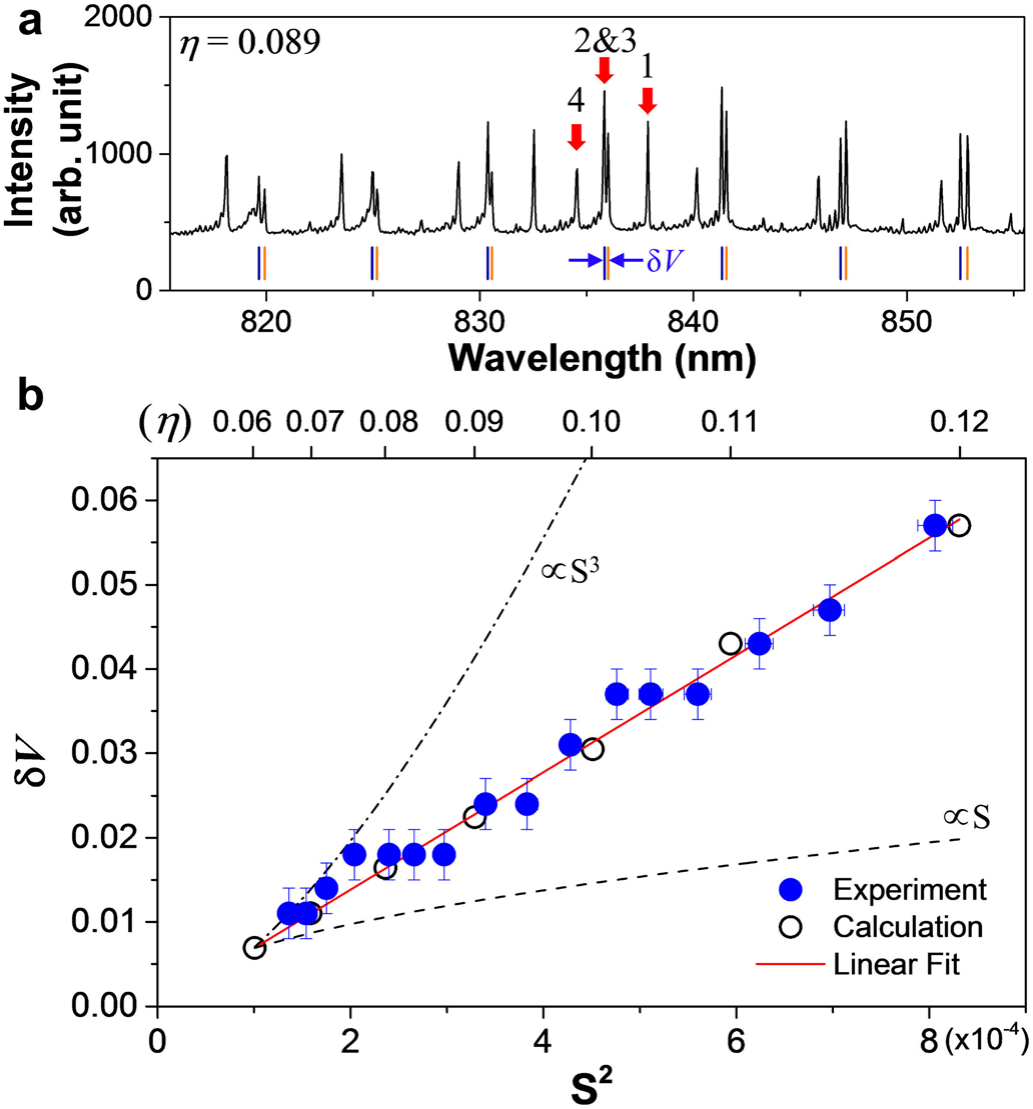
Interaction strength compared with the separatrix area squared. (a) Cavity-modified fluorescence spectrum near *λ* = 835 nm (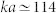) at *η* = 0.089. Peaks corresponding to *l* = 1, 2, 3 and 4 modes are marked by arrows. (b) Separatrix area squared 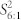 (red solid curve) of the 6:1 resonance structure is compared with the measured AC gaps (blue filled circles) between *l* = 2 and 3 modes near 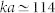 and the ones (black open circles) from wave calculation for various deformation. For comparison, *S*_6:1_ (black dashed) and 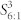 (black dot-dashed) curves are also displayed.

In Fig. 3b, the observed AC gap *δV* (blue-filled circles) is plotted in the unit of the size parameter as a function of the cavity deformation *η* (the upper labels). The decay rates of *l* = 2 and 3 modes, expected to be less than 1 GHz, are negligible compared to the gap size, which is more than 36 GHz, and thus the gap size is approximately twice the coupling strength between *l* = 2 and 3 modes. The nonlinear resonance involved with this mode interaction is 6:1 (*p* = 6, *q* = 1) resonance as illustrated in PSOS in Fig. 1. The PSOS here incorporates the augmented ray dynamics^45^ in order to include the Goos-Hänchen shift coming from the openness of the dielectric cavity. For comparison, the AC gaps (black open circles) from the wave calculation and the values of 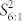 (red solid curve) obtained from the PSOS are also presented in Fig. 3b. We find that our experimental and numerical results well confirm the *S*^2^-dependence of the coupling strength. Interestingly, the AC gaps follow the *S*^2^ curve even in the moderate perturbation regime with 0.10 < *η* < 0.12, where the separatrix shows mild stochasticity. We have not found any intermode interactions violating the relation *δV* ∝ *S*^2^ but satisfying the selection rule.

## Discussion

In order to be able to predict the intermode interaction strength from *S_p_*_:*q*_ by using Eq. (3), we also need to know the proportionality constant or the prefactor *M_p_*_:*q*_. As discussed above, it is not theoretically known for our system. However, we can determine it by the slope of the linear fit in Fig. 3b, where *S*^2^ is obtained from the PSOS presented in a dimensionless (*s*, sin *χ*) phase space. Note we can rescale Eq. (3) as 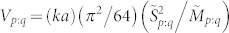 in *ka* unit (Supplementary Note 3), where both 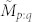 and 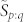 are dimensionless and 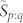 is the separatrix area in the (*s*, sin *χ*) phase space.

**Figure 4:**
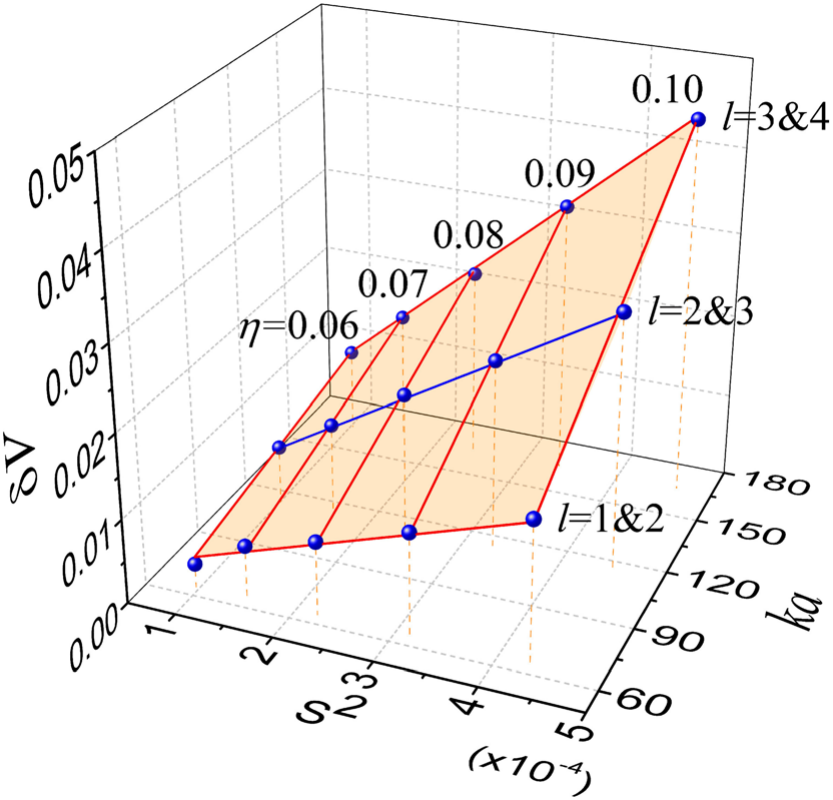
Determining the interaction prefactor. The calculated AC gaps (blue dots) for the interactions between the modes (*l*_1_, *l*_2_) = (1, 2), (2, 3) and (3, 4) for 0.06 < *η* < 0.10 are presented in the *ka* − *S*^2^ space. All of these interactions are mediated by the 6-island resonance structure (*p*:*q*) = 6:1. They are well fit by the rescaled formula in the text to yield a common prefactor 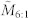 of 0.26 ± 0.01. The experimental data in Fig. 3(b) corresponds to a blue line marked by ‘*l* = 2&3'. The fit surface is a hyperbolic paraboloid.

We found that 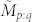 determined by the fitting depends only on the indices (*p*, *q*) of the resonance chain that the interacting modes are associated with. For example, in the range of 0.06 < *η* < 0.10, we numerically observe AC's between *l* = 1 and 2 modes at 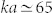, between *l* = 2 and 3 modes at 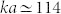 and between *l* = 3 and 4 modes at 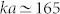, respectively, with all mediated by the same 6:1 resonance chain with the common separatrix area 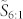. All of these AC gaps are then well fit simultaneously by the above rescaled formula with a common 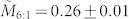 as shown in Fig. 4. The experimental data in Fig. 3b gives 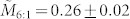. Observation of a common prefactor for those different AC's, although a direct consequence of the RAT theory, is still quite amazing. The fact that the prefactor depends only on the resonance indices allow us to measure the prefactor once and use it for other interactions mediated by the same resonance chain.

As a final remark, it is noted that RAT is usually analyzed in the literature with a dimensionless parameter 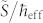 varied. In our work, 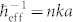 and AC gaps are measured as the cavity deformation and thus 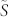 is varied at several different *ka* values as in Fig. 4.

In summary, we have experimentally observed the resonance-assisted tunneling in the intermode interactions in a weak-deformed asymmetric microcavity. A selection rule for the strong interaction mediated by RAT was confirmed. The coupling strength was found to be proportional to the square of the separatrix area of the nonlinear resonance chain involved in the interaction. The prefactor was dependent only on the resonance indices (*p*, *q*). The present findings can be readily applied to other nonintegrable systems, such as nano-electronic devices made of graphene quantum dots corresponding to a two-dimensional quantum billiard^46^,^47^ and a Bose-Einstein condensate under time-dependent perturbations^48^, for analyzing dynamical tunneling and predicting intermode interactions.

## Methods

**Figure 5:**
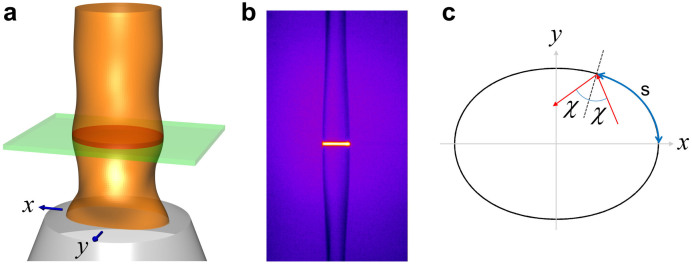
Our fluidic microcavity and Birkhoff coordinates used in obtaining PSOS. (a) Three-dimensional model and (b) an actual microscope image (side view in false color) of our liquid jet column. A few-micron-thick segment at one of the extreme positions of surface modulation is excited by a pump laser to form a two-dimensional microcavity. The cross sectional shape is a quadru-octapole described by a formula in the text. (c) Birkhoff coordinates (*s*, sin *χ*) are used in obtaining the PSOS in Fig. 1. A ray is reflected off the cavity boundary with an incidence angle *χ* at the normalized arc-length coordinate *s*(0 ≤ *s* ≤ 1) along the boundary from the major axis.

Our fluidic microcavity is made of a liquid jet formed by ejecting ethanol vertically through a deformed orifice of a near-elliptical shape. As the liquid column advances, modulation in surface profile spontaneously occurs because the surface tension of liquid acts as a restoring force for an initially noncircular cross section as illustrated in Fig. 5a. A small segment of a few micron thickness of the liquid column at one of extreme positions of surface modulation then acts as a two-dimensional microcavity for the optical wave. The cavity boundary shape can be determined by forward shadow diffraction of a laser beam incident on the jet column^49^, and it is approximately a quadru-octapole given by 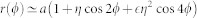, where 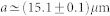 and 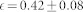. For spectroscopic observations, the liquid contains dye molecules which emit fluorescence when optically excited. When the small segment of the jet column comprising a deformed cavity is excited by a pump laser as seen in Fig. 5b, the fluorescence from dye molecules is enhanced at cavity resonances as shown in Fig. 3a. This enhancement comes from the cavity quantum electrodynamics effect^44^. In this cavity-modified fluorescence spectrum from the microjet cavity, we typically observe 4 ~ 5 groups of cavity resonances or modes. Each mode group is a sequence of resonances with a well-defined free spectral range.

The PSOS in Fig. 1 is presented in Birkhoff coordinates (*s*, sin *χ*), where a ray is reflected off the cavity boundary at the normalized arc-length coordinate *s*(0 ≤ *s* ≤ 1) along the boundary from the major axis with an incidence angle *χ* as illustrated in Fig. 5c. For each reflection we employed the augmented ray dynamics^45^ in order to include the Goos-Hänchen shift. For the PSOS in Fig. 1, *ka* = 115 is assumed.

## Author Contributions

H.K., J.Y., S.-B.L. and K.A. conceived the experiment. H.K. performed the experiment, analyzed the data and carried out theoretical investigations. K.A. supervised overall experimental and theoretical works. H.K., Y.S., S.M. and K.A. wrote the manuscript. All authors participated in discussions.

## Supplementary Material

Supplementary Information Supplementary Information

## Figures and Tables

**Table 1:** Comparison between the observed angular mode number difference Δ*m* and the resonance index *p* (the number of islands) of the resonance chain structure involved in various intermode interactions in our ADM

l	interaction	Δm	p	Δm = np
2 vs. 3	strong	6	6	satisfied
3 vs. 4	strong	6	6	satisfied
1 vs. 2	weak	8	8	satisfied
2 vs. 4	weak	12	6	satisfied
1 vs. 3	weak	14	6	not satisfied
1 vs. 4	weak	20	8	not satisfied
