# Supplementary material for: Nonlinear resonance-assisted tunneling induced by microcavity deformation
Source: Sci Rep. 2015 Mar 11;5:9010. doi: 10.1038/srep09010 (PMC4355734; doi:10.1038/srep09010)
Supplement: Supplementary Information [file srep09010-s1.pdf]

Nonlinear resonance-assisted tunneling induced by microcavity deformation

Hojeong Kwak,[1,*] Younghoon Shin,[1] Songky Moon,[1]
Sang-Bum Lee,[2] Juhee Yang,[3] and Kyungwon An[1,†]

[1]*School of Physics and Astronomy, Seoul National University, Seoul 151-742, Korea*

[2]*Korea Research Institute of Standards and Science, Daejeon 305-340, Korea*

[3]*Russia Science Seoul, Korea Electrotechnology Research Institute, Seoul 121-912, Korea*

[*]Present address: Samsung Electronics Company, Hwaseong 445-330, Korea

[†]Electronic address: kwan@phya.snu.ac.kr

SUPPLEMENTARY INFORMATION

I. RESONANCE-ASSISTED TUNNELING IN A 2D SYSTEM

In this section, we recapitulate the resonance-assisted tunneling (RAT) theory for a two-dimensional (2D) system in the framework of the standard secular perturbation theory and discuss its applicability to a 2D optical microcavity.

The Hamiltonian dynamics near nonlinear resonances can be described by using the secular perturbation theory [1, 2]. For a 2D system, the Hamiltonian can be decomposed as

$$H = H_0(I_1, I_2) + V(I_1, I_2, \theta_1, \theta_2), \tag{S1}$$

in terms of action-angle variables. Here H_0 is an integrable Hamiltonian and V, which is a small perturbation, may contain nonintegrable terms. A resonance arises when the condition

$$p\frac{dH_0}{dI_1} = q\frac{dH_0}{dI_2}. \tag{S2}$$

is satisfied for co-prime positive integers p and q. With a choice of a generating function

$$F_2 = (\theta_1 - \frac{q}{p}\theta_2)\hat{I}_1 + \theta_2\hat{I}_2, \tag{S3}$$

we obtain canonical transformation from $(I_1, I_2, \theta_1, \theta_2)$ to $(\hat{I}_1, \hat{I}_2, \hat{\theta}_1, \hat{\theta}_2)$ as

$$\begin{aligned} I_1 &= \hat{I}_1, \\ I_2 &= \hat{I}_2 - \frac{q}{p}\hat{I}_1, \\ \hat{\theta}_1 &= \theta_1 - \frac{q}{p}\theta_2, \\ \hat{\theta}_2 &= \theta_2. \end{aligned} \tag{S4}$$

For this transformation, $\hat{\theta}_1$ remains constant at the $p : q$ resonance and varies slowly near the resonance whereas $\hat{\theta}_2$ varies rapidly there. It is then possible to "average" the Hamiltonian over θ_2 in order to obtain a transformed Hamiltonian to the first-order,

$$H_{p:q} = H_0 + \bar{V}(\hat{I}_1, \hat{I}_2, \hat{\theta}_1), \tag{S5}$$

where $\bar{V} = \frac{1}{2\pi p}\int_0^{2\pi p} d\theta_2 V(I_1, I_2, \theta_1, \theta_2)$. Since $\bar{V}$ is a $\frac{2\pi}{p}$-periodic function of $\hat{\theta}_1$, it can be expanded in series as

$$\bar{V} = \sum_{i=0} V_{p:q}^{(i)} \cos(ip\hat{\theta}_1 + \xi_i). \tag{S6}$$

Note $V_{p:q}^{(i)}$ generally falls off rapidly as i increases, so we can take only the leading terms with $i = 0, 1$. In addition, without loss of generality we can take $\xi_1 = 0$ by trivial constant shift in $\hat{\theta}_1$.

By expanding H_0 around $I_{p:q}$, the action at the resonance, to the non-vanishing lowest power of $(I - I_{p:q})$, ignoring the constant terms in H_0 and $\bar{V}$ and keeping the lowest-order terms in the perturbation, we can derive a pendulum-like effective Hamiltonian near the $p : q$ resonance as

$$H_{p:q} = \frac{(I - I_{p:q})^2}{2M_{p:q}} + V_{p:q} \cos p\theta, \tag{S7}$$

where we have substituted $I = \hat{I}_1$, $\theta = \hat{\theta}_1$ and $V_{p:q} = V_{p:q}^{(1)}$. We have also defined

$$M_{p:q}^{-1} \equiv \left. \frac{d^2 H_0}{dI^2} \right|_{I=I_{p:q}} . \tag{S8}$$

For two-dimensional billiard systems, the Hamiltonian can be described in the polar coordinate (r, θ). For a circular boundary shape, the angular coordinate θ and its conjugate momentum $\hbar ka \sin\chi$ are the action-angular (θ_1, I_1) whereas the radial coordinate r and its conjugate momentum are not action-angle variables. However, the radial coordinate can be transformed to an angle variable θ_2 in principle [3], because the motion in circular billiard is “periodic” in the radial coordinate, although we do not have to find that transformation for deriving Eq. (S7). Therefore, the formulation presented above can be readily applied to 2D billiard systems.

II. UNPERTURBED-BASIS MODES

Unperturbed-basis modes (UBM’s) in a weakly deformed microcavity are referred to the eigenstates of a fictitious Hamiltonian H' associated with the actual Hamiltonian H of the system. Under UBM description, the fictitious Hamiltonian H' is diagonalized with its diagonal elements the same as those of H while the off-diagonal elements of H correspond to the coupling between UBM’s. In the mode dynamics diagram shown in Fig. 2 in the main text, UBM’s follow the diabatic lines denoted by dashed lines. Some eigenstates (or modes) of the actual Hamiltonian H exhibit avoided crossings when they get near with each other because of the off-diagonal elements under UBM description. An actual eigenstate can then be expressed as a linear superposition of UBM’s.

III. RELATION BETWEEN THE GAP OF AVOIDED CROSSING AND THE PHASE-SPACE AREA S

Let us consider the trajectories of Eq. (S7) in the $(\theta - I)$ phase space. When $H_{p:q} = V_{p:q}$, the trajectory lies on the separatrix given by

$$I - I_{p:q} = \pm\sqrt{2M_{p:q}V_{p:q}}\sqrt{1-\cos p\theta} \tag{S9}$$

Then the area $S_{p:q}$ enclosed by the separatrix is given by

$$\begin{aligned} S_{p:q} &= 2\int_0^{2\pi}(I - I_{p:q})d\theta \\ &= 2p\sqrt{2M_{p:q}V_{p:q}}\int_0^{2\pi/p}\sqrt{1-\cos p\theta}d\theta \\ &= 2\sqrt{2M_{p:q}V_{p:q}}\int_0^{2\pi}\sqrt{1-\cos x}dx \\ &= 16\sqrt{M_{p:q}V_{p:q}}. \end{aligned}$$

and thus

$$V_{p:q} = \frac{S_{p:q}^2}{256M_{p:q}}. \tag{S10}$$

By using this relation, we can infer the magnitude of the perturbation $V_{p:q}$ if we know the phase-space area $S_{p:q}$.

In a 2D optical microcavity, ray motion is conveniently described in the Birkhoff coordinates $(s, \sin\chi)$, where s is the normalized arc length $(0 \le s \le 1)$ along the boundary and χ is the incident angle of a ray at s with respect to the boundary normal. The action variable can be written as $I = \hbar ka\sin\chi = \hbar ka\tilde{I}$, where $\tilde{I} = \sin\chi$, the dimensionless momentum component in the Birkhoff coordinate, and k the wave vector. The effective Hamiltonian of Eq. (S7) is then rewritten in terms of dimensionless variables as

$$\tilde{H} = \frac{(\tilde{I} - \tilde{I}_{p:q})^2}{2\tilde{M}_{p:q}} + \tilde{V}_{p:q}\cos 2\pi ps. \tag{S11}$$

where $\tilde{H} \equiv H/(\hbar kc), \tilde{V}_{p:q} \equiv V_{p:q}/(\hbar kc), \tilde{I}_{p:q} \equiv I_{p:q}/(\hbar ka)$ and $\tilde{M}_{p:q} \equiv M_{p:q}/(\hbar ka^2/c)$. In this $(s - \tilde{I})$ phase space, the relation Eq. (S10) is scaled to be

$$\tilde{V}_{p:q} = \left(\frac{\pi^2}{64}\right)\left(\frac{\tilde{S}_{p:q}^2}{\tilde{M}_{p:q}}\right). \tag{S12}$$

The area $\tilde{S}_{p:q}$ is readily obtained from the Poincarè surface of section in the Birkhoff coordinates.

In our experiment the gap of avoided crossing (AC) is measured in terms of size parameter ka. For high Q modes, the AC gap is approximately given by twice of the coupling strength g between two UBM's. Since an UBM $|m\rangle$ with angular quantum number m has the angular dependence $e^{i2\pi ms}$, the coupling strength g between m and $m+p$ modes is given by

$$\begin{aligned} g &= \langle m|V_{p:q}\cos 2\pi ps|m+p\rangle \\ &\simeq V_{p:q}\int_0^1 e^{-i2\pi ms}\cos 2\pi ps\ e^{i2\pi(m+p)s}ds \\ &= \frac{V_{p:q}}{2}. \end{aligned}$$

The AC gap is then given by

$$(\text{AC gap}) \simeq 2g \simeq V_{p:q} = \hbar kc\tilde{V}_{p:q}. \tag{S13}$$

In ka unit, we let $\hbar c = a$, and thus

$$(\text{AC gap}) \simeq (ka)\left(\frac{\pi^2}{64}\right)\left(\frac{\tilde{S}_{p:q}^2}{\tilde{M}_{p:q}}\right). \tag{S14}$$

Exact calculation of $\tilde{M}_{p:q}$ requires knowledge on the integrable Hamiltonian H_0. Unfortunately, the exact form of H_0 is not known in general. In our work, $\tilde{M}_{p:q}$ is obtained from the data fitting with Eq. (S14). It shows that the AC gaps of modes associated with the same resonance structure $(p:q)$ differ only by their ka values since they share the same $\tilde{M}_{p:q}$ and $\tilde{S}_{p:q}$. This feature elucidates the semiclassical nature of the resonance-assisted tunneling.

The interaction Hamiltonian has higher-order cosine terms like $\cos(ip\theta)$ as shown in Eq. (S6), so the UBM $|m\rangle$ can couple to $|m+ip\rangle$ mode with a coupling constant proportional to $\tilde{V}_{p:q}^{(i)}$ or $(\tilde{V}_{p:q})^i$. Both amplitudes are much smaller than $\tilde{V}_{p:q}$. This is why the AC gap between $l=2$ and 4 modes with $\Delta m = 12 = 2p$ (second order) in Fig. 1 in the text appear much weaker than that of $l=2$ and 3 (or $l=3$ and 4) with $\Delta m = p = 6$ (first order).

IV. SPATIAL MODE DISTRIBUTIONS AND HUSIMI FUNCTIONS OF THE MODES MARKED AS (I)-(IV) IN FIG. 2

We solved the wave equation for the same size and shape as our liquid-jet microcavity by employing the boundary element method (BEM) [4]. The resulting quasi-eigenvalues or

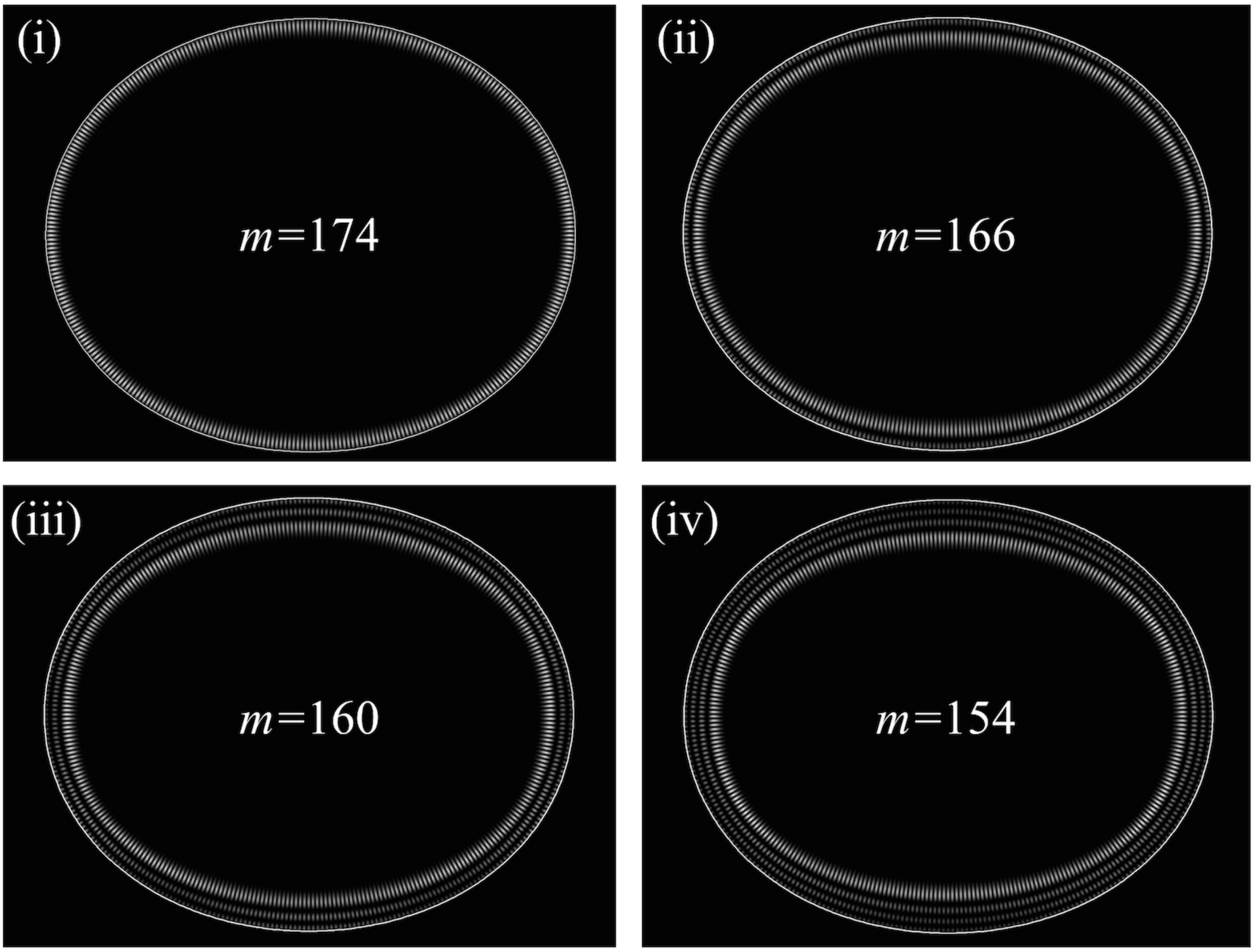

FIG. S1: **Intensity plots of the spatial mode distributions.** When $\eta = 0.10$, spatial mode distributions are shown for l=1, 2, 3 and 4 modes marked by (i)-(iv), respectively, in the uncoupled region around $ka \sim 133$ in Fig. 2. The solid line indicates the cavity boundary.

resonance-mode frequencies are presented in terms of the size parameter ka with $k = 2\pi/\lambda$ the wavevector and a the mean radius of the cavity. Spatial intensity plots of the l=1, 2, 3 and 4 modes marked by (i), (ii), (iii) and (iv) in Fig. 2 are shown in Fig. S1. They are far from each other in resonance frequency, *i.e.*, located in an uncoupled region. Their husimi functions are shown in Fig. S2. The angular mode number m is just the half of the number of antinodes in Fig. S1. The radial mode number l is the same as the number of anti-nodes in the radial direction. We can identify the resonance chain (thus p) involved in the interaction

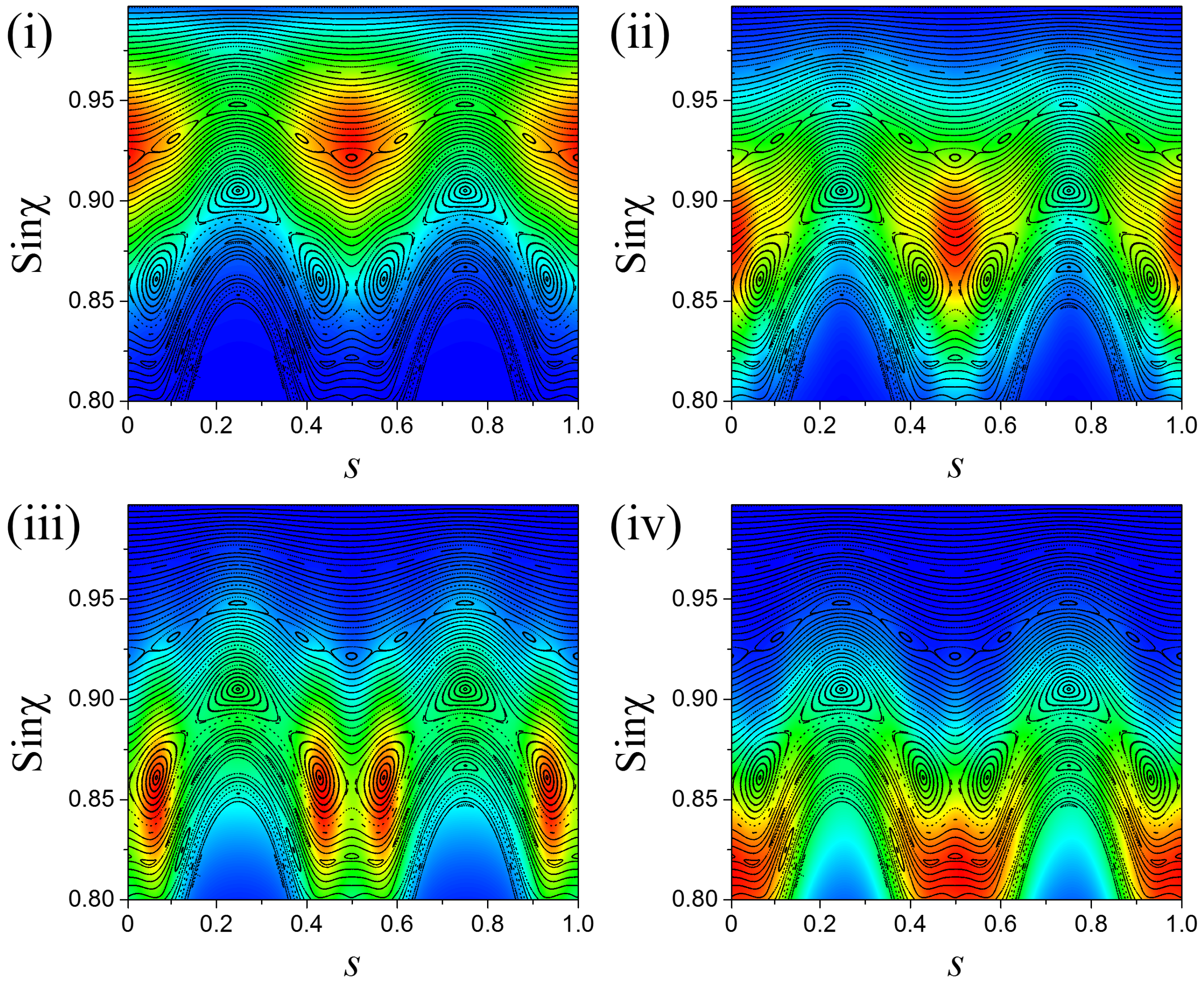

FIG. S2: **Husimi functions.** When $\eta = 0.10$, phase-space distributions or Husimi functions are shown for the modes marked by (i)-(iv) in the uncoupled region around $ka \sim 133$ in Fig. 2. (i) $l = 1$ mode is located above the $p = 8$ resonance chain. (ii) $l = 2$ mode is located below the $p = 8$ resonance chain but above the $p = 6$ resonance chain. (iii) $l = 3$ mode is located at or just below $p = 6$ resonance chain. (iv) $l = 4$ mode is located much below $p = 6$ resonance chain. As ka increases, the position of these modes shift upward gradually so that $l = 3$ mode become located just above $p = 6$ resonance chain when $150 < ka < 180$ within the ka range of our investigation.

by comparing the PSOS and the husimi functions of the involved modes in Fig. S2.

---

[1] A. M. Ozorio de Almeida, J. Phys. Chem. **88**, 6139 (1984).

[2] A. J. Lichtenberg and M. A. Liebermann, *Regular and Stochastic Motion* (Springer, New York, 1983).

[3] H. Goldstein, C. P. Poole and J. L. Safko, *Classical Mechanics, 3rd edition* (Addison Wesley, San Francisco, 2002)

[4] J. Wiersig, J. Opt. A: Pure Appl. Opt. **5**, 53 (2003).
